# Genome-wide characterization of simple sequence repeats in *Pyrus bretschneideri* and their application in an analysis of genetic diversity in pear

**DOI:** 10.1186/s12864-018-4822-7

**Published:** 2018-06-18

**Authors:** Huabai Xue, Pujuan Zhang, Ting Shi, Jian Yang, Long Wang, Suke Wang, Yanli Su, Huirong Zhang, Yushan Qiao, Xiugen Li

**Affiliations:** 1 College of Horticulture, Nanjing Agricultural University, Nanjing, 210095 China; 2 Zhengzhou Fruit Research Institute, Chinese Academy of Agricultural Sciences (CAAS), Key Laboratory of Fruit Breeding Technology of Ministry of Agriculture, Zhengzhou, 450009 China

**Keywords:** *Pyrus bretschneideri*, Simple sequence repeat, Genetic diversity, Pear

## Abstract

**Background:**

Pear (*Pyrus* spp.) is an economically important temperate fruit tree worldwide. In the past decade, significant progress has been made in pear molecular genetics based on DNA research, but the number of molecular markers is still quite limited, which hardly satisfies the increasing needs of geneticists and breeders.

**Results:**

In this study, a total of 156,396 simple sequence repeat (SSR) loci were identified from a genome sequence of *Pyrus bretschneideri* ‘Dangshansuli’. A total of 101,694 pairs of SSR primers were designed from the SSR loci, and 80,415 of the SSR loci were successfully located on 17 linkage groups (LGs). A total of 534 primer pairs were synthesized and preliminarily screened in four pear cultivars, and of these, 332 primer pairs were selected as clear, stable, and polymorphic SSR markers. Eighteen polymorphic SSR markers were randomly selected from the 332 polymorphic SSR markers in order to perform a further analysis of the genetic diversity among 44 pear cultivars. The 14 European pears and their hybrid materials were clustered into one group (European pear group); 29 Asian pear cultivars were clustered into one group (Asian pear group); and the Zangli pear cultivar ‘Deqinli’ from Yunnan Province, China, was grouped in an independent group, which suggested that the cultivar ‘Deqinli’ is a distinct and valuable germplasm resource. The population structure analysis partitioned the 44 cultivars into two populations, Pop 1 and Pop 2. Pop 2 was further divided into two subpopulations. Results from the population structure analysis were generally consistent with the results from the UPGMA cluster analysis.

**Conclusions:**

The results of the present study showed that the use of next-generating sequencing to develop SSR markers is fast and effective, and the developed SSR markers can be utilized by researchers and breeders for future pear improvement.

**Electronic supplementary material:**

The online version of this article (10.1186/s12864-018-4822-7) contains supplementary material, which is available to authorized users.

## Background

Pear (*Pyrus* spp., 2n = 2× = 34) is an economically important temperate fruit tree worldwide. The genus *Pyrus* belongs to the subtribe Malinae of the tribe Maleae in the subfamily Amygdaloideae of the family Rosaceae [1], with three known secondary centers of origin: the Chinese center, the Central Asian center, and the Near Eastern center [2].

Because pear is self-incompatible and has a long juvenile period, the traditional breeding method based only on general appearance and agronomic performance is time consuming and money intensive. With the development of molecular breeding technology, marker-assisted selection, an important tool now in the improvement of many crops, permits the rapid identification of key individuals that harbor useful genes and offers promise for pear breeding. Providing as many molecular markers as possible in the whole genome is the prerequisite for the construction of a genetic linkage map, gene mapping, molecular marker assisted selection (MAS) and other genomics studies [3–5].

Microsatellites, or simple sequence repeats (SSRs), are tandemly repeated units of 1–6 nucleotide sequence motifs flanked by unique sequences [6–8]. Given their wide distribution throughout the genome, codominant inheritance, and high polymorphism [7, 9, 10], SSRs have become desirable molecular markers for the construction of genetic linkage maps [11], genetic relationship identification [12], fingerprinting [13] and genetic diversity analyses [14–17].

Currently, several efforts have also been made to develop SSR markers in pears [17–26]. Yamamoto et al. [21–23] developed SSR markers, which showed a high degree of polymorphism, in Japanese pear (*Pyrus pyrifolia* Nakai) for the first time by using an enriched genomic library, RAHM (random amplified hybridization microsatellites) and 5′ anchored PCR methods. Fernández-Fernández et al. developed 19 microsatellite primer pairs from the genomic DNA of European pear (*Pyrus communis* L.) [19]. Nishitani et al. developed 73 expressed sequence tag (EST)-simple sequence repeat (SSR) markers based on ESTs derived from 11 cDNA libraries of the Japanese pear cultivar ‘Housui’, and the SSR markers showed good transferability to other species in the Rosaceae [20]. Yamamoto et al. developed 237 SSR markers from a genome sequencing analysis in Japanese pear, and most of them successfully added to genetic linkage maps of pear [24]. Fan et al. reported a set of 120 SSRs that was developed from the newly assembled pear sequence and evaluated polymorphisms in seven genotypes of pear from different genetic backgrounds [18]. Chen et al. [11] developed 1341 SSR markers based on the genome sequence of the ‘Dangshansuli’ pear [27]. There are also many SSR loci from transcriptomes of different tissues of pear that have been identified and reported [17, 25, 26].

Although a great deal of effort has been made for the development of SSRs based on the genome sequence [11, 15, 18] since the genome of *Pyrus bretschneideri* Rehd was completely sequenced [27], the number of SSRs publicly available for pear is still insufficient for some applications, such as the construction of high-resolution linkage maps, QTL mapping analyses, increasing marker density in specific map regions and so on. Therefore, more efforts are still needed to develop SSR markers for further progress in pear genetics and genomics studies. In the present study, we aimed to: (1) develop SSR markers from publicly available scaffold sequence of the pear genome; (2) anchor the SSR markers to the existing reference genetic map; and (3) evaluate SSR polymorphism and applications in the analysis of genetic diversity.

## Results

### Identification and distribution of SSRs in the genome

A total of 156,396 SSR motifs were identified within the scaffold sequences. Of the total SSRs identified, mono-nucleotide repeat motifs (98,939, 63.26%) were predominant, followed by di-nucleotide repeat motifs (41,546, 26.56%), tri-nucleotide repeat motifs (11,798, 7.54%), tetra-nucleotide repeat motifs (3271, 2.09%), penta-nucleotide repeat motifs (593, 0.38%) and hexa-nucleotide repeat motifs (249, 0.16%). For the mono-, di- and tri-nucleotide repeat motifs, the frequency is as high as 97.36% in total. The most abundant mono-nucleotide motif was A/T, accounting for 95.94% of mono-nucleotide motif repeats. In di-nucleotide repeats, the most frequent motif was AG/CT (42.34%), followed by AT/TA (40.36%). Of the tri-nucleotide repeats, AAG/CTT and AAT/ATT were the most abundant, accounting for 27.61 and 26.45%, respectively. The number of each major SSR type identified is summarized in Additional file 1: Figure S1; Additional file 2: Table S1 and Additional file 3: Table S2.

Further comparison of the number of SSRs with different repeat motifs revealed that the number of SSRs with shorter motifs was much higher than that with longer motifs. In all repeat types, the number of SSRs exceeded 10,000 only for the mono-nucleotide repeat motifs with the repeat numbers of 12, 13 and 14. The number of SSRs for the mono-, di-, tri-, and tetra-nucleotide repeat motif types were more than 1000 when the repeat numbers was within 28, 17, 7 and 5, respectively, while the number of SSRs was not more than 500 for the penta- and hexa-nucleotide repeat motifs even with the least number of repeats (Additional file 4: Table S3).

### Functional annotation of SSR loci

By searching for genomic annotation documents (GFF), among the 156,396 SSR loci identified, there are 120,469 SSR loci that were located within or near the 28,819 genes. Among them, the number of SSRs in the intergenic region was 62,984 (40.27%), which was the highest, followed by the number of SSRs located in the intron of the gene (20,085, 12.84%). There are 23,953 (15.32%) SSRs in total located within the 1 KB area upstream of a transcription start site, within the 1 KB area downstream of a transcription start site, or within a 1 KB area upstream of a gene while downstream of another gene. There are 5052 SSRs in total in the region of 5’ UTR, or in the region of 3’ UTR. Only 2135 SSRs were located in the exon region of coding genes (Additional file 5: Table S4).

**Fig. 1:**
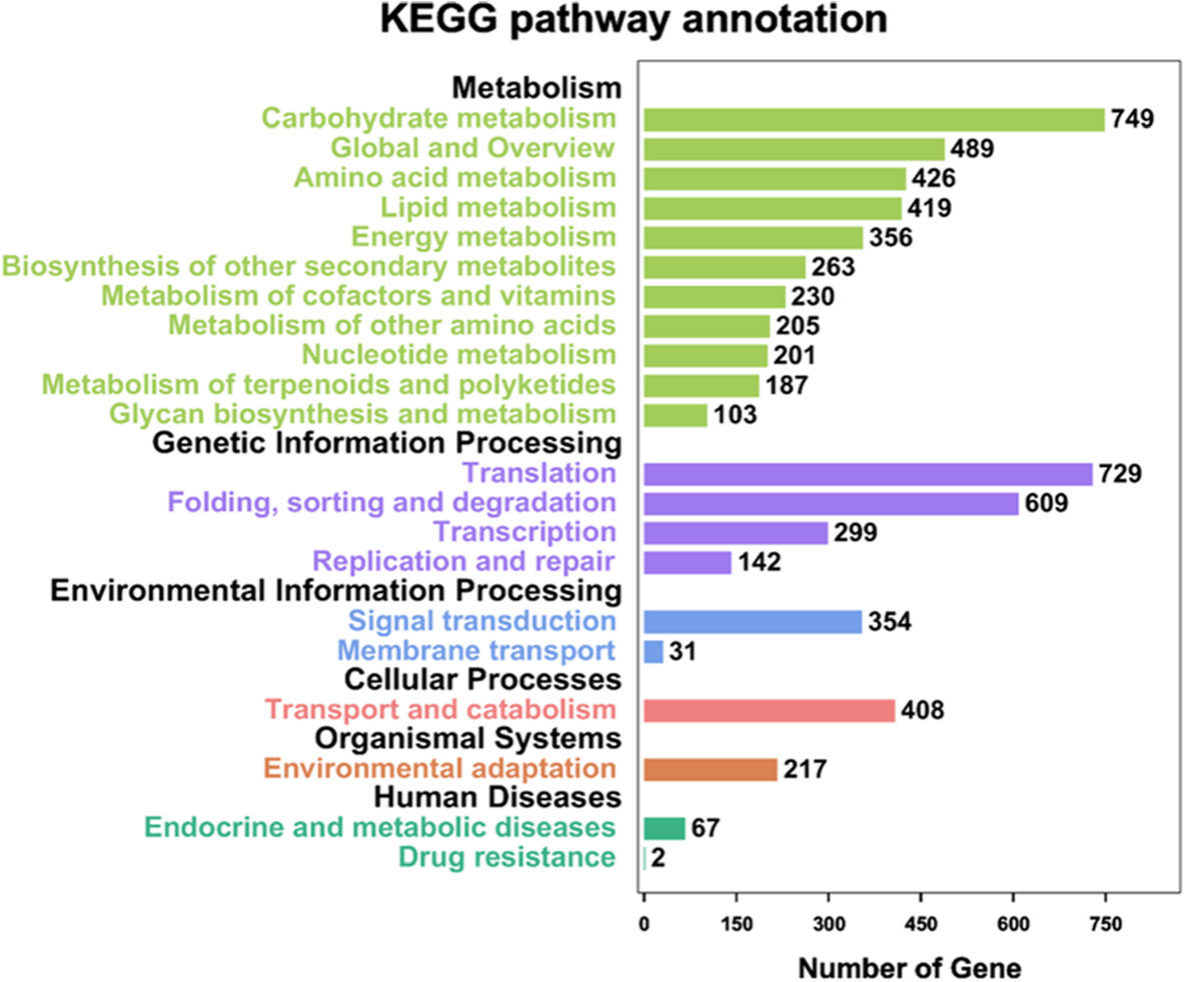
Result of the KEGG pathway annotation

All the 28,819 genes associated with the 120,469 SSRs loci were searched using BLASTx against the National Central for Biotechnology Information (NCBI) database, and 24,333 genes were annotated within the database. These genes were also aligned by BLASTx to the protein database KEGG, and 4835 genes were annotated within the KEGG database. These genes were enriched into 128 pathways of 6 KEGG A classes and 21 KEGG B classes. With the 6 KEGG A classes, 417 genes were enriched into Cellular Processes, 385 genes were enriched into Environmental Information Processing, 1990 genes were enriched into Genetic Information Processing, 69 genes were enriched into Human Diseases, 4674 genes were enriched into Metabolism, and 217 genes were enriched into Organismal Systems (Fig. 1).

**Fig. 2:**
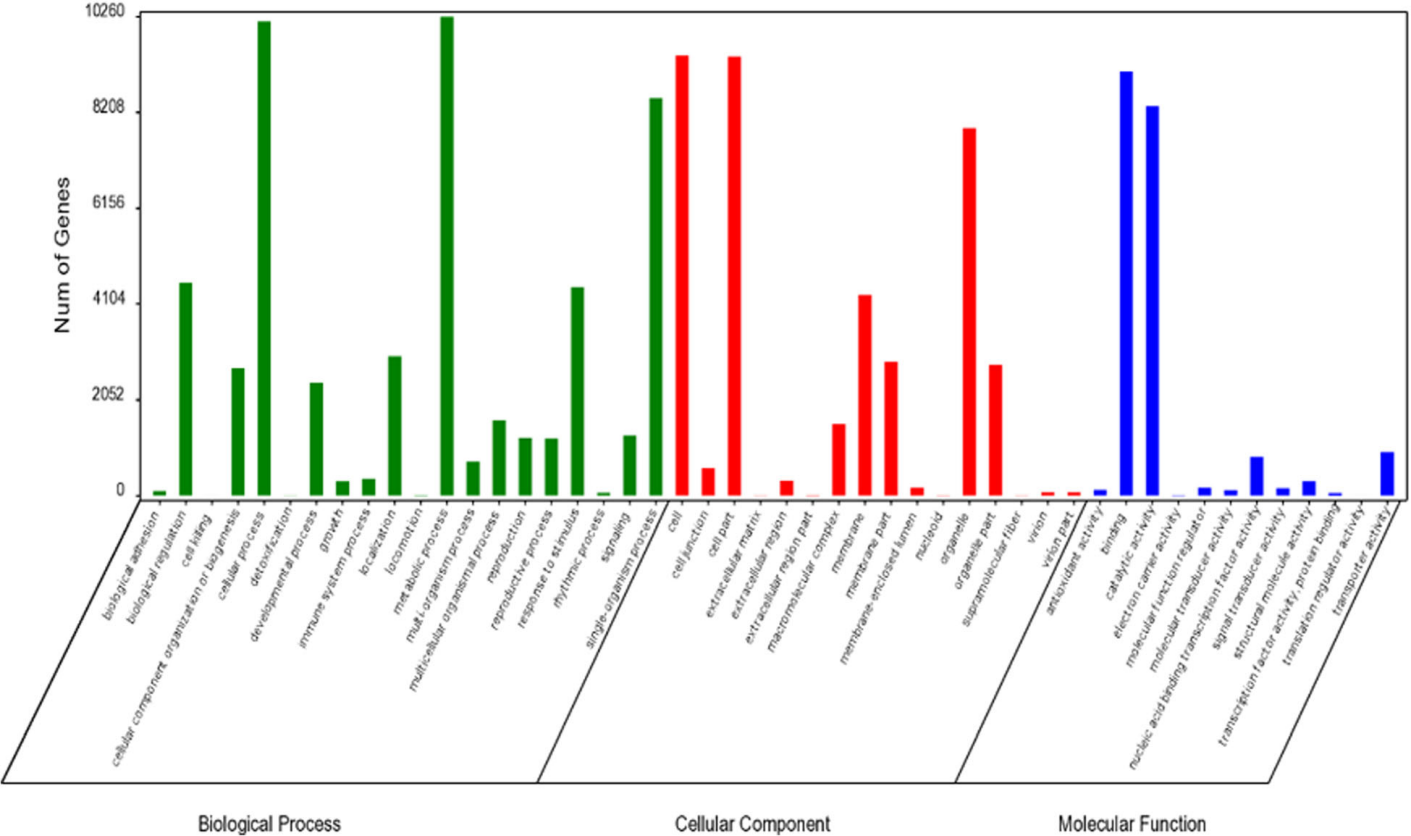
GO terms of the genes

GO functional annotations of 28,819 genes were analyzed by the Blast2GO software (version 2.6.0+) and classified further using GO terms. After acquiring a GO annotation for every gene, we used WEGO to perform a GO functional classification for all the genes. Based on the GO annotation, 15,808 (54.85%) genes of pear were assigned to 112,706 GO-term annotations. These annotations were summarized into 48 GO-terms of the three major categories. Of them, biological process (53,051, 47.07%) comprised the majority of the GO annotations, followed by cellular component (39,492, 35.04%) and molecular function (20,163, 17.89%). Genes were mostly enriched in the terms of metabolic process (10257), cellular process (10158), cell (9421), cell part (9405) and binding (9088) (Fig. 2).

### Development of SSR primers and determination of their linkage groups

A total of 153,008 (97.83%) primer pairs were designed from the 156,396 identified SSRs. After deletion of the SSR loci with more than one identical primer, a total of 101,694 SSR loci (Additional file 6: Table S5, 65.02%) were eventually retained, including 63,317 (62.26%) mono-nucleotide repeat motifs, 27,561 (27.10%) di-nucleotide repeat motifs, 8261 (8.12%) tri-nucleotide repeat motifs, 1962 (1.93%) tetra-nucleotide repeat motifs, 409 (0.40%) penta-nucleotide repeat motifs and 184 (0.18%) hexa-nucleotide repeat motifs (Additional file 3: Table S2).

According to the data of the high-density genetic map of pear [28], we designated the SSR loci to the corresponding linkage group (LG) and provided the scaffolds’ genetic position to the SSR loci derived from the scaffolds as an estimated reference position in the linkage group. Of the 101,694 SSR loci, a total of 61,160 SSR loci were localized to a single linkage group. The 19,255 SSR loci may be located in one of the 2–3 linkage groups, but the specific linkage group could not be identified. A total of 21,279 SSR loci could not be located on a specific linkage group, and the broad linkage group could not be determined. Of the 17 linkage groups, the SSR loci were the least in the seventh linkage group, 1011 in total; followed by first linkage group, with 1491 SSR loci; and the fifteenth linkage group had the largest number of SSR loci, 7030 in total (Additional file 6: Table S5; Additional file 7: Table S6).

### Polymorphism of SSR primers

**Fig. 3:**
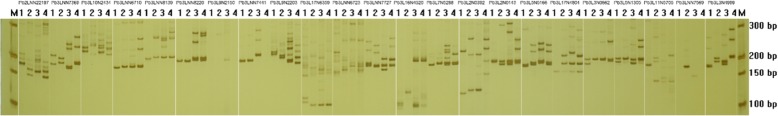
PAGE results from the PCR amplification of 23 SSR primer pairs at a 60 °C annealing temperature. M: DL2000 DNA Ladder Marker (TaKaRa); 1: Red Clapp’s Favorite; 2: Mansoo; 3: Mantianhong; 4: Hongxiangsu

A total of 534 SSR primer pairs were tested for their ability to amplify DNA and detect polymorphism in 4 cultivars using a PAGE analysis (Fig. 3; Additional file 8: Table S7). Among them, 27 SSR primer pairs failed to amplify the product (5.06%), and the rest of the 507 SSR primer pairs could amplify the expected product (94.94%). Through further screening optimization, we screened out 332 (62.17%) SSR primer pairs that have clear banding patterns and are easy to identify (Additional file 9: Table S8). They can be used not only in the genetic population analysis but also in germplasm identification and a genetic diversity analysis.

### Assessment of the genetic relationship among 44 pear cultivars by SSR markers

To verify the applicability of the newly developed SSR primer, a total of 44 pear varieties were genotyped by 18 SSR primers. The results showed that 18 pairs of primers amplified a product with a size range of 103–275 bp (two markers as examples, see Additional file 10: Figure S2). Most of the actual size of the amplified fragments matched or was very close to the size of the expected fragments; only the amplified products of primer Pb2L17N16884 were different from the expected fragment size. The 18 pairs of SSR primers detected 5–15 alleles in the tested cultivars, with an average allele number of 10.56. All the alleles were grouped into 11–26 genotypes, with an average genotype number of 19.22. The observed heterozygosity (No) among the SSR primers ranged from 0.2826 (Pb3LUN6782) to − 0.8913 (Pb2L17N16884), with an average No of 0.6763. The polymorphism information content (PIC) ranged from 0.6064–0.8814, with an average PIC of 0.7808 (Additional file 11: Table S9).

**Fig. 4:**
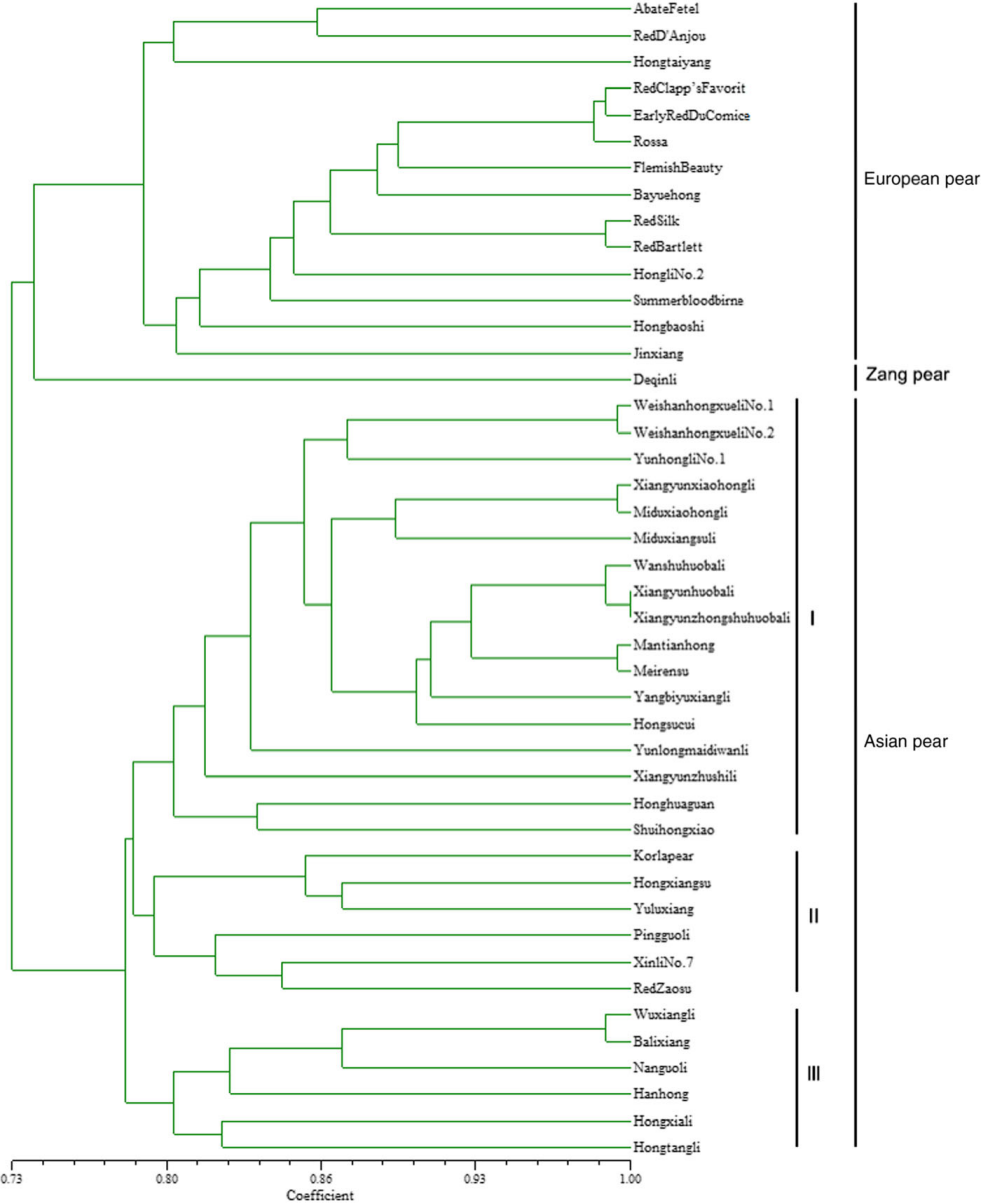
Dendrogram for 44 pear cultivars derived from the UPGMA cluster analysis of 18 polymorphic SSR markers

In the UPGMA cluster analysis, the 44 pear accessions were classified into three groups (For similarity coefficient among 44 pear cultivars, see Additional file 12: Table S10). The ‘Deqinli’ of the Zangli pear is grouped into one group individually, which is in a special position in the dendrogram. Among the other 43 varieties, 14 pear cultivars, including 9 European pear accessions and 5 hybrids derived from European pears, were grouped into one group, and 29 Asian pear varieties were clustered into one group (Fig. 4).

The Asian pear group can be further divided into 3 subgroups: the first subgroup is mainly composed of Chinese sand pear (*P. pyrifolia*) from Yunnan Province, China, and the offspring (‘Mantianhong’, ‘Meirensu’, ‘Hongsucui’) of the Yunnan sand pear cultivar ‘Huobali’. Cultivar ‘Shuihongxiao’ of white pear and cultivar ‘Honghuaguan’ of *Pyrus ussuriensis* from Hebei Province, China are also clustered in this group. The second subgroup is composed of ‘Korla pear’, ‘Pingguoli’ and the descendants of ‘Pingguoli’, such as ‘Hongxiangsu’, ‘Yuluxiang’, ‘Xinli No.7’ and ‘Red Zaosu’, which can be called the Chinese white pear (*P. bretschneideri*) subgroup. The third subgroup is composed of ‘Wuxiangli’ and other 6 varieties of *Pyrus ussuriensis* (Fig. 4).

**Fig. 5:**
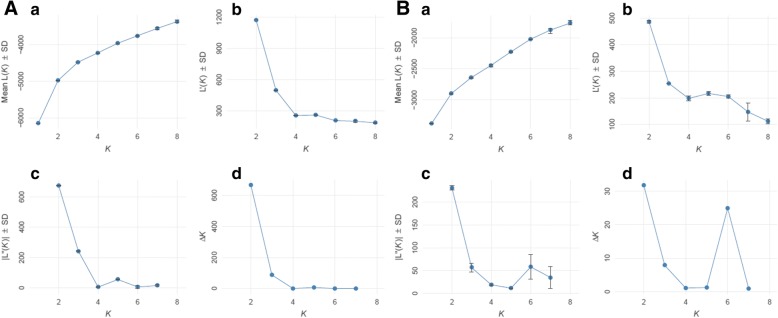
Evanno analysis for 44 varieties (**A**) and 29 Oriental pear varieties (**B**) with (*a*) estimated log probability of data of runs over increasing values of K, (*b*) first derivative, (*c*) second derivative and (*d*) ΔK over values of K

**Fig. 6:**
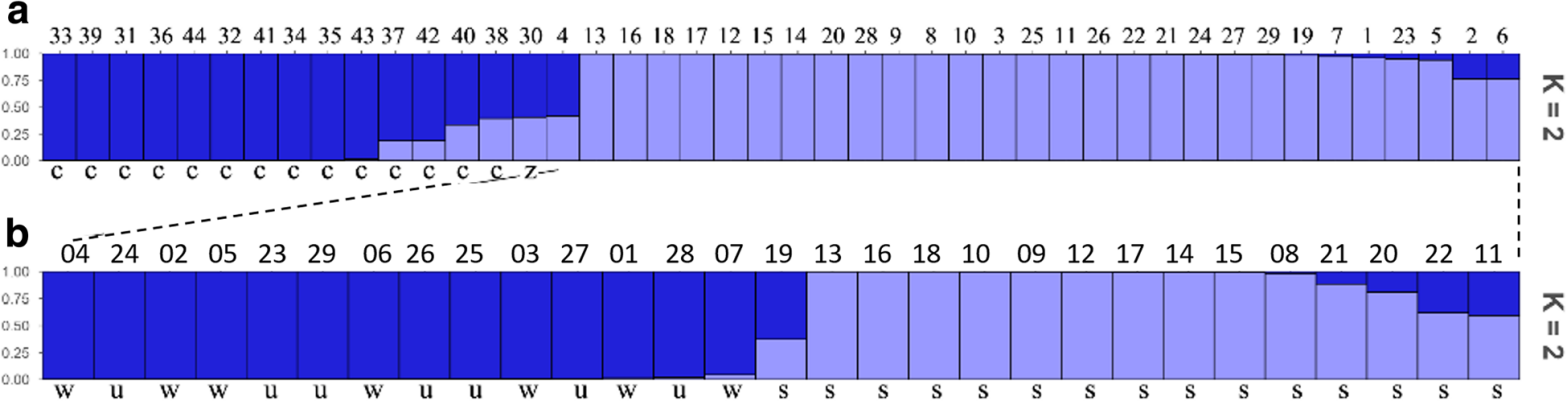
The structure analysis of 44 pear accessions (**a**) and 29 Asian pear accessions (**b**). c: *P. communis*, z: Zangli, w: *P. bretschneideri* (white pear), u: *P. ussuriensis,* s: *P. pyrifolia* (sand pear)

In the population structure analysis, there is an obvious turning point for the estimated log probability of data when K = 2 (Fig. 5A-a). The first derivative (Fig. 5A-b), second derivative (Fig. 5A-c) and the peak value of the ΔK score (Fig. 5A-d) were found to be the greatest when K = 2, suggesting that the 44 accessions were partitioned into two groups corresponding to the European pear group (Pop 1) and the Asian pear group (Pop 2) (Fig. 6a). The 15 accessions in Pop 1 and 29 accessions in Pop 2 were further analyzed using the same methods, and only Pop 2 was subdivided into two subpopulations (Fig. 5B). Subpopulation 1 included 15 accessions comprising all the 6 accessions from the second subgroup, all the 6 accessions from the third subgroup, and 3 accessions from the first group (Fig. 6b). In comparison, subpopulation 2 mainly comprised 14 out of 17 accessions from the first group obtained in the UPGMA cluster analysis (Fig. 6b). The results from the population structure analysis were generally consistent with the results from the UPGMA cluster analysis.

## Discussion

SSRs can be divided into genomic SSRs and genic SSRs, and genomic SSRs are usually derived from SSR-enriched genomic libraries or random genomic sequences, while genic SSRs are derived from coding regions of the transcriptome or EST sequences [17, 26, 29]. Compared with genomic SSR markers, genic SSR markers are considered to link to the loci of agronomic phenotypes and are more useful for marker assisted selection (MAS), especially when polymorphic genic SSR markers were identified in breeding lines [29, 30]. Although genic SSRs are more effective, genome-wide mining of genomic SSRs is also a fundamental work. The availability of more and more whole-genome sequences for an increasing number of species will also greatly facilitate the development of genomic SSRs, and some research has also shown that SSRs in introns or other noncoding regions are also strongly associated with phenotypes [31, 32]. Pan et al. [32] found that genotypes of a TC microsatellite in an intron of *PpYUC11* had strong association with the flesh texture (normal or stony hard) phenotypes of 43 peach varieties, and they considered that *PpYUC11* might be responsible for the stony hard phenotype in peach. In other research, Kang et al. [31] reported that a chimeric mRNA (*Psy1/Unknown*), which was generated by joining exons from *Psy1* and an unknown gene and resulted in the yellow-fruited phenotype of tomato accession PI 114490, might be caused by an SSR with 19 A/T repeats in the downstream sequence of the *Psy1* gene with *Psy1/Unknown*. A recent study shows that intron DNA sequences constitute a previously unrecognized type of downstream regulatory element for genes transcribed by RNA polymerase II and play an important role in determining the site of transcription initiation [33]. Clearly, the genomic SSRs located in introns and in the downstream sequence of genes may have the same enormous potential as genic SSRs in MAS applications. In the present study, a total of 49,384 SSR loci were identified from the intron and 1 KB upstream and downstream from the transcription initiation site, and a total of 7917 SSR loci were identified from the extrons, 5’ UTR and 3’ UTR, which demonstrates the importance of the development of SSR markers in the whole genome.

The functions of genic SSRs can often be inferred by performing a homology comparison of gene-containing genic SSRs, which is one of the reasons researchers think genic SSRs are more useful than genomic SSRs [29, 30]. With the development of next-generation sequencing (NGS) technology, many species have had their whole genome sequenced, and the development of genomic SSR markers on a genome-wide scale using genomic sequencing data has gradually become an option of research [34–38]. The functions of the genomic SSRs developed from the related species can also be inferred from the gene annotation files or the function of neighboring genes. On the other hand, the genic SSRs developed from a specific transcriptome have a number of limitations due to the specificity of gene expression [26], while the genic SSRs developed from different material or tissue transcriptome data will compensate for this limitation to some extent, but it also increases the redundancy of genic SSRs, which has caused inconvenience to the application of genic SSR markers. Of course, genomic SSRs developed in early stages using genomic libraries or random DNA sequences also have the disadvantage of unclear location information and redundant loci, which are not conducive to applications.

In the present study, the 18 SSR loci distinguished the 44 pear individuals with an average of 10.56 alleles per locus and an average PIC value of 0.7808, indicating a high level of polymorphism. Comparison of our results with previous findings of the SSR-based studies in Pear revealed that the average number of alleles per locus and PIC values recorded in the present study were compatible or higher than other SSR-based studies in *Pyrus*. Yue et al. [17] evaluated 28 *Pyrus* accessions with 28 genic SSR markers and identified 9.43 alleles per locus and an average PIC value of 0.585. Liu et al. [39] studied the group structure of 45 European pears (*Pyrus communis* L.) using 134 core SSR markers and showed that the average number of alleles per SSR locus was 5.45. Erfani et al. [40] studied the genetic diversity of 47 pear cultivars and genotypes using 28 SSR markers and showed that the average number of alleles per SSR locus was 6.21. Song et al. [15] in the study of genetic variability of 99 *P. pyrifolia* cultivars detected a mean of 4.93 alleles per locus for 134 SSR markers varying from 3 to 9. Brini et al. [41] reported the average number of alleles per locus in pear ranged from 4 to 10, with an average of 7. Average PIC values obtained in our study were also somewhat higher when compared to the results of other authors. Wolko et al. [42] found PIC values were in the range of 0.42 to 0.89 with an average of 0.65 for the 19 high polymorphic SSR markers selected from 40 tested SSR markers. Dequigiovanni et al. [43] reported PIC values for 62 SSR loci among 42 pear accessions ranged from 0.48 to 0.87, with an average of 0.67. Yue et al. [17] reported an average PIC value of 0.58 for 28 SSRs in 28 *Pyrus* accessions (0.26 to 0.91). Erfani-Moghadam and Zarei [44] found PIC values of the SSR markers varied from 0.44 to 0.69 with an average of 0.59. Thus, the randomly selected 18 SSR markers showed a high level of polymorphism. Some of the SSR markers developed in the present study have also been applied to construction of high-resolution linkage maps and QTL mapping analyses in pear by Wang et al. [45] and Wang et al. [46], respectively, which also proved advantageous to the application. The SSR markers developed in the present study are the most abundant pear SSR loci to date, and also provided the physical location and the estimated genetic positions for reference, which could significantly increase the efficiency of the related genomics studies, such as genetic diversity, constructing high-resolution linkage maps, QTL mapping, and so on.

In the present study, we used the UPGMA method to cluster the 44 pear varieties, and the European pears and Asian pears were clustered into different groups. A similar distinct clustering pattern has also been reported, and the Asian pear and European pear were completely separated from each other in the phylogenetic tree [17, 47] and probably evolved independently [48]. For the three subgroups of Asian pear, the phylogenetic tree showed that the relationship between subgroup I of sand pear and subgroup II of white pear is close. The subgroup of sand pear cultivars consisted of sand pear cultivars and the white pear cultivar ‘Shuihongxiao’, which have not been completely separated. This result is consistent with previous studies [13, 17, 47, 49] and proved that the correctness of the white pear cultivar group was assigned as a subgroup of sand pear of the *P. pyrifolia* white pear group [50]. In the previous studies, *P. ussuriensis* cultivars were considered to have originated from a hybrid between wild *P. ussuriensis* and *P. bretschneideri* [51], so *P. ussuriensis* cultivars were sometimes clustered alone [14] and sometimes clustered together with sand pear or white pear [13, 50]. In the present study, we also obtained similar results, i.e., the *P. ussuriensis* cultivar ‘Honghuaguan’ was not clustered with the *P. ussuriensis* cultivars in subgroup III, but first with the white pear cultivar ‘Shuihongxiao’ and then clustered with the 15 sand pear cultivars as subgroup I of Asian pear.

Huobali is a group of local cultivars in Yunnan Province, China. ‘Wanshuhuobali’, ‘Xiangyunhuobali’ and ‘Xiangyunzhongshuhuobali’ are closely related cultivars to each other, all of which were clustered in subgroup I of Asian pear. ‘Mantianhong’, ‘Hongsucui’ and ‘Meirensu’, the descendants of their male parent Huobali (probably ‘Anninghuobali’), were also clustered together with these three cultivars. Also in subgroup II, ‘Hongxiangsu’, ‘Xinli No.7’ and ‘Yuluxiang’, all of which were offspring of ‘Korla pear’, were clustered together with ‘Korla pear’. Also grouped with these breeds are ‘Xinli No.7’ (filial generation of ‘Korla pear’ and ‘Zaosu’), ‘RedZaosu’ (bud mutation of ‘Zaosu’) and ‘Pingguoli’ (female parent of ‘Zaosu’). That these relatives were clustered together showed that the clustering results in this study were relatively reliable.

Zangli pear, a variety of landrace, distributed in the higher altitude junction region of Tibet, Yuannan and Sichuan Provinces in southwestern China, is a kind of semi-wild or semi-cultivated type, with hardy characteristics and resistance to a hypoxic environment. This type of pear is a potential germplasm resource available for resistance breeding in pear; for example, Zangli varieties ‘Deqinli’ pear, distributed in Deqin County of the Diqing Tibetan Autonomous Prefecture in Yunnan Province, China, have morphological characteristics of both Oriental and European pear, but its genetic relationship is closer to European pear, with smaller and leathery leaves. In the present study, the results of a cluster analysis of 44 pear cultivars with newly developed SSR markers also showed that the phylogenic status of ‘Deqinli’ in the dendrogram was rather special, indicating that it was closer to European pear. Another study also showed that the relationship between Zangli and sand pear cultivars from Sichuan, Yunnan and Guizhou Provinces was closer than that between Zangli and *P. ussuriensis* cultivars, but the European pears were not included in the study [52]. There are also some semi-cultivated pear cultivars with both Asian and European pear characteristics in Xinjiang Province, Northwest China. Among them, the variety ‘Wanshu Duxiaxi’ was clustered together with some European Pear cultivars, such as ‘Batllet’, ‘La France’ and ‘Passe Crassane’, into one group [47], indicating the genetic background of these semi-cultivated varieties have high similarities with European pear. To determine the relationships between these pear germplasm resources in Southwest China and Northwest China, and the relationship between them and other pear systems, further studies are still needed. This will help us better understand the origin and evolution of *Pyrus*, and at the same time, can help us make use of these resources.

## Conclusions

In this study, we developed 101,694 genomic SSR markers from the reference genome sequences of pear. Among them, a total of 38,377 SSR marker loci were 2–6 base repeats, which is the largest number of SSR markers developed from a single development in pear by far. These SSR markers were also marked with the relative position information of both the physical location and the linkage groups, and the adjacent genes of the SSR loci were also annotated, which can provide better help for related research work and the developed SSR markers can be utilized by researchers and breeders for future pear improvement.

## Methods

### Plant materials and DNA extraction

A total of 44 accessions, planted in the Pear Germplasm Resources Garden in Zhengzhou, Zhengzhou Fruit Research Institute, Chinese Academy of Agricultural Sciences, were used in the present study (Tables 1 and 2). XGL is responsible for a formal and more detailed description of each of these pear accessions. The voucher specimens for these pear accessions used herein are deposited in Zhengzhou Fruit Research Institute, Chinese Academy of Agricultural Sciences, China. No specific permissions were required for these plant material, since these studies did not involve endangered or protected species. Young leaves of different accessions were collected in April 2016.

**Table 1:** The 44 accessions used in the polymorphism analysis

No.	Accession	Identification No.	Parentage	Origin	Species
1	Hongxiangsu	ZFRpyA9–13	Korla pear× Eli	Henan, China	*P. bretschneideri*
2	Pingguoli	ZFRpyA12–10	Unknown	Jilin, China	*P. bretschneideri*
3	Yuluxiang	ZFRpyA10–13	Korla × Xuehua	Shanxi, China	*P. bretschneideri*
4	Red Zaosu	ZFRpyA18–3	Red mutation of Zaosu	Shan’xi, China	*P. bretschneideri*
5	Korla pear	ZFRpyA10–6	Unknown	Xinjiang, China	*P. bretschneideri*
6	Xinli No.7	ZFRpyA9–14	Korla pear × Zaosu	Xinjiang, China	*P. bretschneideri*
7	Shuihongxiao	ZFRpyA15–2	Unknown	Hebei, China	*P. bretschneideri*
8	Hongsucui	ZFRpyA1–4	Kousui × Huobali	Henan, China	*P. pyrifolia*
9	Mantianhong	ZFRpyA1–8	Kousui × Huobali	Henan, China	*P. pyrifolia*
10	Meirensu	ZFRpyA2–2	Kousui × Huobali	Henan, China	*P. pyrifolia*
11	Miduxiangsuli	ZFRpyB14–17	Unknown	Yunnan, China	*P. pyrifolia*
12	Miduxiaohongli	ZFRpyB14–20	Unknown	Yunnan, China	*P. pyrifolia*
13	Wanshuhuobali	ZFRpyB14–19	Unknown	Yunnan, China	*P. pyrifolia*
14	Weishanhongxueli No.1	ZFRpyB14–32	Unknown	Yunnan, China	*P. pyrifolia*
15	Weishanhongxueli No.2	ZFRpyB14–15	Unknown	Yunnan, China	*P. pyrifolia*
16	Xiangyunhuobali	ZFRpyB14–28	Unknown	Yunnan, China	*P. pyrifolia*
17	Xiangyunxiaohongli	ZFRpyB14–16	Unknown	Yunnan, China	*P. pyrifolia*
18	Xiangyunzhongshuhuobali	ZFRpyB14–38	Unknown	Yunnan, China	*P. pyrifolia*
19	Xiangyunzhushili	ZFRpyB14–18	Unknown	Yunnan, China	*P. pyrifolia*
20	Yangbiyuxiangli	ZFRpyB14–33	Unknown	Yunnan, China	*P. pyrifolia*
21	Yunhongli No.1	ZFRpyA1–17	Unknown	Yunnan, China	*P. pyrifolia*
22	Yunlongmaidiwanli	ZFRpyB14–34	Unknown	Yunnan, China	*P. pyrifolia*
23	Hanhong	ZFRpyA14–1	Nanguoli × Jinsu	Jilin, China	*P. ussuriensis*
24	Nanguoli	ZFRpyA15–4	Unknown	Liaoning, China	*P. ussuriensis*
25	Balixiang	ZFRpyA17–10	Unknown	Liaoning, China	*P. ussuriensis*
26	Wuxiangli	ZFRpyA14–13	Unknown	Liaoning, China	*P. ussuriensis*
27	Hongtangli	ZFRpyB14–3	Unknown	Hebei, China	*P. ussuriensis*
28	Honghuaguan	ZFRpyB14–6	Unknown	Hebei, China	*P. ussuriensis*
29	Hongxiali	ZFRpyB14–14	Unknown	Hebei, China	*P. ussuriensis*
30	Deqinli	ZFRpyB16–12	Unknown	Yunnan, China	Zangli
31	Early Red Du Comice	ZFRpyA15–7	Unknown	Britain	*P. communis*
32	Red Bartlett	ZFRpyB17–22	Bartlett mutation	America	*P. communis*
33	Red Clapp’s Favorite	ZFRpyA16–15	Clapp’s Favorite mutation	America	*P. communis*
34	Red D’Anjou	ZFRpyA17–18	Beurre D’Anjou mutation	America	*P. communis*
35	Summer blood birne	ZFRpyA16–7	Unknown	America	*P. communis*
36	Flemish Beauty	ZFRpyA16–3	Unknown	Belgium	*P. communis*
37	Abate Fetel	ZFRpyA15–8	Unknown	France	*P. communis*
38	Hongbaoshi	ZFRpyD2–1	Bayuehong × Dangshan suli	Henan, China	*P. communis*
39	Rossa	ZFRpyA15–17	Unknown	Italy	*P. communis*
40	Jinxiang	ZFRpyB17–1	Bartlett × Nanguoli	Liaoning, China	*P. communis*
41	Bayuehong	ZFRpyB17–20	Clapp’s Favorite × Zaosu	Shan’xi, China	*P. communis*
42	Hongli No.2	ZFRpyA18–5	Qiyuesu × Red Clapp’s Favorite	Henan, China	*P. communis*
43	Hongtaiyang	ZFRpyA17–14	Clapp’s Favorite × Xinglongmali	Henan, China	*P. communis*
44	Red Silk	ZFRpyB17–16	Unknown	America	*P. communis*

**Table 2:** Eighteen polymorphic SSR markers validated in the 44 pear accessions

Marker	Repeat motif	Primer sequence (5′ → 3′)	Ta (°C)	Expected length (bp)	Actual length (bp)
Pb3L11N5758	(aat)14	F: GGTGCTGTAAGGTGGTGATG R: ATGAAACCAGCCAAACAACC	58	224	183–225
Pb3L4N5130	(aat)16	F: GCCTCCAGACTCAAACTTTCC R: TGCGAGAGCGTAGGAGAAAT	56	229	205–244
Pb2LUN25693	(ac)23	F: CTTCGAAAGGTTCCCAGAAG R: AAGTACGGACCAAGGTGCAG	53	200	169–225
Pb2L10N09474	(ac)23	F: CATAAGCACCCAAACTCGTG R: GCCATGAGTCAAAGAGTCCAG	58	244	208–250
Pb3L15N3832	(aca)10	F: AATAGCGCATAGATCCAACCA R: CCTCCCCATAAGCTTCATCA	58	203	183–207
Pb3LUN6782	(aca)13	F: TGCTTTACAGCTGAGCTTCG R: AAGTTTCCCTCGGGGTTTTA	58	213	184–211
Pb2L15N13836	(ag)20	F: CGTCTTGAACCACCATCTCC R: CCACTTTCTTCCACCACCAT	56	199	173–211
Pb2L2N00815	(ag)20	F: CCAAACACATGACCACAAGC R: AAATGGAGCAGGTGGGAGTA	56	192	164–198
Pb2L10N09198	(ag)20	F: GAGAAATGCAGTGGGGATTG R: AAAGTGACCGCTCAAAATGC	56	205	179–207
Pb2LUN24134	(ag)21	F: AAGCTGGTTATGTGGCTGCT R: ATTGAGTTCGCTCGTTCGTT	56	204	171–197
Pb2L17N16884	(ag)21	F: TTGTGCCCTTTTTCCTACCA R: GGGCTAAACGCTTTGATGTT	58	197	107–147
Pb2L8N07068	(ag)21	F: AGGAGCTAGCATGCTTTGGA R: CGACACGAACACAAGAAACA	56	192	103–275
Pb2L1N00036	(ag)22	F: CACTATGCCAGTGACAAAGATTG R: AGTAGATGCTAGGGCCACCA	58	198	174–214
Pb2L7N06609	(ag)22	F: ACCCTAGTCGTCGTTCTTGC R: GCAGAGACGGAAAGAAATGG	56	207	149–207
Pb2L4N03431	(ag)22	F: CCCAAAAGTAACCGAGCGTA R: TTCAGCCAGCCACTCTCTCT	56	191	168–200
Pb3L2N0143	(aga)12	F: ACCAGAACCCACTCACCATC R: CCGACAATAAAGGCCTCAA C	56	198	178–199
Pb3L5N5166	(aga)12	F: CGAGAGCGCCTAAGAGAAGA R: CGGAGACTCGCTGACTCACT	58	211	182–209
Pb4L1N0014	(cata)11	F: TCCCTTCAAGGCTGTGAGTT R: CAGAGGTTTGGTTTTGGTGA	58	211	172–208

Genomic DNA was extracted using the modified CTAB (cetyltrimethy-lammonium bromide) method. The DNA quality and concentration were determined by electrophoresis in 1% agarose gel and NanoDrop1000 spectrophotometry (NanoDrop, Thermo scientific, Wilmington, DE, USA). DNA were diluted with sterilized ultrapure water, normalized to 50 ng/μL, and stored at − 20 °C until use.

### The identification and annotation of SSR motifs

Scaffold sequences in a compressed file “225117_ref_Pbr_v1.0_chrUn.fa” for the genome of ‘Dangshansuli’ pear were obtained through ftp://ftp.ncbi.nlm.nih.gov/genomes/Pyrus_x_bretschneideri/CHR_Un/ [27]. SSR motifs were identified using a Perl language script Msatfinder [53]. A minimum repeat threshold of twelve, eight, five, five, five, and five repeats were required for detecting mono-, di-, tri-, tetra-, penta- and hexa-nucleotide motifs, respectively.

We carried out the SSR annotation from the reference genome with the Generic Feature Format 3 (GFF3) (ftp://ftp.ncbi.nlm.nih.gov/genomes/Pyrus_x_bretschneideri/GFF/) using the ANNOVAR software [54]. To annotate the genes, we used the BLASTx (version 2.3.5) program (https://blast.ncbi.nlm.nih.gov/Blast.cgi) with an E-value threshold of 1e-5 to the NCBI non-redundant protein (Nr) database (http://www.ncbi.nlm.nih.gov) and the Kyoto Encyclopedia of Genes and Genomes (KEGG) database (http://www.genome.jp/kegg). Protein functional annotations could then be obtained according to the best alignment results. The GO annotation of genes was analyzed by the Blast2GO software (version 2.6.0+) [55], and the functional classification of genes was completed using WEGO software [56].

### Primer design and marker validation

Based on SSR motifs, the Primer 3 software [57] was used to design SSR primers with the following parameters: the length of primers was in the range of 18–28 bp, with 20 bp as the optimum; the product size range was from 100 to 300 bp; and the melting temperature (Tm) was in the range of 55–65 °C, with 60 °C as the optimum and a maximum Tm difference of 1 °C. Primers of the SSRs with compound motif types were discarded. All the primer sequences were compared with each other, and the primers with more than one copy were deleted to verify that each primer pair amplified only a single SSR. These newly developed scaffold-derived SSR markers were then aligned to the high-density genetic linkage map [28] according to the scaffolds’ genetic positions, which were defined as the average genetic position of the SNPs on a scaffold of the map. Each primer pair was named with the prefix code Pb (*Pyrus bretschneideri*), followed by the repeated motif type, a linkage group number and a consecutive number.

A total of 534 primers were randomly selected from the newly designed primers to first validate for polymerase chain reaction (PCR) and SSR polymorphisms among four pear accessions (Red Clapp’s Favorite, Hongsucui, Mantianhong and Hongxiangsu). The PCR reaction was carried out in a reaction mixture with a volume of 20 μL comprised of 1.0 μL (50 ng) of template DNA, 2.0 μL of 10 × PCR buffer (Mg^2+^ plus), 0.1 μL of *Taq* DNA polymerase solution (5 unit/μL), 1.6 μL of 25 mM dNTPs, 0.6 μL (10 μM) of each primer solution and 14.1 μL of sterilized ddH_2_O. The PCR reagents (buffer, MgCl_2_, dNTPs and *Taq*) were purchased from TaKaRa Bio Engineering Co., Ltd. (Dalian, China), and the primers were synthesized by GENEWIZ Bioengineering Ltd. (Beijing, China). Amplification was programmed as 5 min at 94 °C for initial denaturation; 30 cycles consisting of 30 s at 94 °C for denaturation, 30 s at the annealing temperature (Ta) for annealing, 45 s at 72 °C for extension; and finally, a 10-min extension step at 72 °C. The PCR products were loaded for electrophoresis in an 8% polyacrylamide gel.

Based on the screening results, 18 polymorphic primer pairs were further used to genotype the 44 pear accessions using the same volume and PCR program. Forward primers of the 18 primer pairs were 5′ end labeled with a fluorescent dye (HEX or FAM). The PCR products were identified on an ABI 3130 genetic analyzer (Applied Biosystems, Foster City, CA, USA). Determination of the fragment sizes and data collection were done using the GeneMapper 4.0 software (Applied Biosystems, Foster City, CA, USA).

### Data analysis

The genotyping data of the accessions in the 18 SSR loci selected in the initial screening were then used to evaluate the genetic diversity of the 44 pear accessions. To estimate the genetic diversity among the pear accessions, some parameters were calculated using the software POPGENE version 1.32 [58], including the number of alleles (Na), number of genotypes (Ng), major allele frequency (A), observed heterozygosity (Ho), and expected heterozygosity (He). The polymorphism information content (PIC) of each locus was calculated using the software PowerMarker version 3.25 [59]. The software program NTSYS-pc2.10 was used to conduct a cluster analysis using the UPGMA method [60]. Population structure was analyzed and visualized using R package POPHELPER [61] and STRUCTURE 2.3.4 [62].

## Additional files

Additional file 1: **Figure S1.** Percentages of different motifs among mono-(a), di- (b) and tri- (c) nucleotide repeats in the ‘Dangshansuli’ pear genome. (PDF 183 kb)

Additional file 2: **Table S1.** Frequency of different SSR repeat motifs. (XLSX 9 kb)

Additional file 3: **Table S2.** Frequency distribution of different SSR motifs. (XLSX 9 kb)

Additional file 4: **Table S3.** Type and number of repeat motifs. (XLSX 10 kb)

Additional file 5: **Table S4.** Statistics of SSRs in different categories. (XLSX 9 kb)

Additional file 6: **Table S5.** Number of different types of SSR motifs in different LGs. (XLSX 10 kb)

Additional file 7: **Table S6.** Information of new designed 101, 694 SSR primers, including information of the primer sequences, annealing temperature (Ta), repeat motifs, target size, linkage groups, and positions in genetic and physical maps of pear. (XLSX 10192 kb)

Additional file 8: **Table S7.** Information of 534 SSR primers, including information of the primer sequences, annealing temperature (Tm), repeat motifs, target size, linkage groups, and positions in genetic and physical maps of pear. (XLSX 72 kb)

Additional file 9: **Table S8.** Information of 332 good SSR primers, including information of the primer sequences, annealing temperature (Tm), repeat motifs, target size. (XLSX 36 kb)

Additional file 10: **Figure S2.** Amplified fragments of Pb3L11N5758 and Pb3L4N5130 SSR loci from five pear varieties. (PDF 188 kb)

Additional file 11: **Table S9.** Polymorphism information of 18 primers in 44 pear cultivars. (XLSX 10 kb)

Additional file 12: **Table S10.** Similarity coefficient among 44 pear cultivars. (XLSX 18 kb)
