# Supplementary figures and images for: Genome-wide characterization of simple sequence repeats in Pyrus bretschneideri and their application in an analysis of genetic diversity in pear
Source: BMC Genomics. 2018 Jun 18;19:473. doi: 10.1186/s12864-018-4822-7 (PMC6006662; doi:10.1186/s12864-018-4822-7)

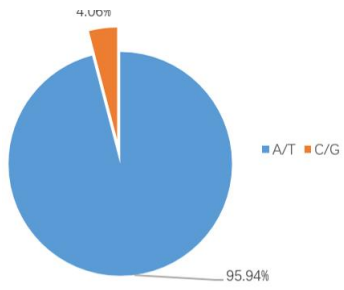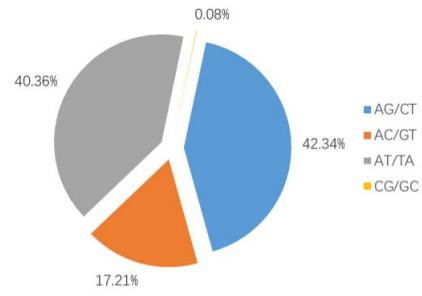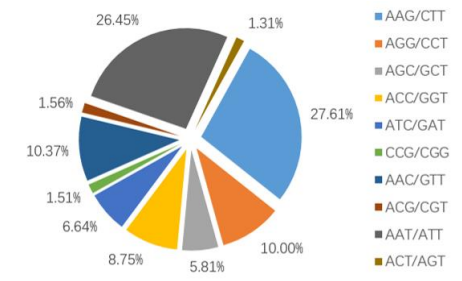

Supplement: Supplementary file 1 — Figure S1. Percentages of different motifs among mono-(a), di- (b) and tri- (c) nucleotide repeats in the ‘Dangshansuli’ pear genome. (PDF 183 kb) [file 12864_2018_4822_MOESM1_ESM.pdf]

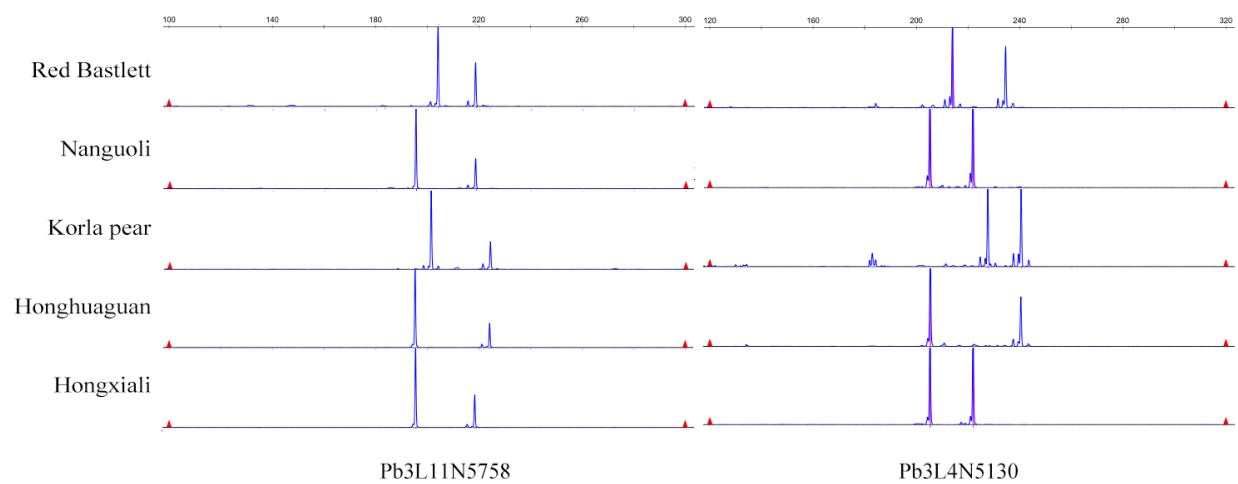

Supplement: Supplementary file 10 — Figure S2. Amplified fragments of Pb3L11N5758 and Pb3L4N5130 SSR loci from five pear varieties. (PDF 188 kb) [file 12864_2018_4822_MOESM10_ESM.pdf]
